# Altered patterns of fractional amplitude of low-frequency fluctuation and regional homogeneity in abstinent methamphetamine-dependent users

**DOI:** 10.1038/s41598-021-87185-z

**Published:** 2021-04-08

**Authors:** An Xie, Qiuxia Wu, Winson Fu Zun Yang, Chang Qi, Yanhui Liao, Xuyi Wang, Wei Hao, Yi-Yuan Tang, Jianbin Liu, Tieqiao Liu, Jinsong Tang

**Affiliations:** 1 Department of Radiology, Hunan Provincial People’s Hospital, First Affiliated Hospital of Hunan Normal University, Changsha, 410005 China; 2 National Clinical Research Center for Mental Disorders, and Department of Psychiatry, The Second Xiangya Hospital of Central South University, Changsha, Hunan 410011 China; 3 Department of Psychological Sciences, Texas Tech University, Lubbock, TX USA; 4 Department of Psychiatry, Zhejiang Provincial People’s Hospital, People’s Hospital of Hangzhou Medical College, Hangzhou, China; 5 Department of Psychiatry, Sir Run Run Shaw Hospital, School of Medicine, Zhejiang University, Hangzhou, Zhejiang China

**Keywords:** Neuroscience, Medical research

## Abstract

Methamphetamine (MA) could induce functional and structural brain alterations in dependent subjects. However, few studies have investigated resting-state activity in methamphetamine-dependent subjects (MADs). We aimed to investigate alterations of brain activity during resting-state in MADs using fractional amplitude of low-frequency fluctuation (fALFF) and regional homogeneity (ReHo). We analyzed fALFF and ReHo between MADs (n = 70) and healthy controls (HCs) (n = 84) and performed regression analysis using MA use variables. Compared to HCs, abstinent MADs showed increased fALFF and ReHo values in the bilateral striatum, decreased fALFF in the left inferior frontal gyrus, and decreased ReHo in the bilateral anterior cingulate cortex, sensorimotor cortex, and left precuneus. We also observed the fALFF values of bilateral striatum were positively correlated with the age of first MA use, and negatively correlated with the duration of MA use. The fALFF value of right striatum was also positively correlated with the duration of abstinence. The alterations of spontaneous cerebral activity in abstinent MADs may help us probe into the neurological pathophysiology underlying MA-related dysfunction and recovery. Since MADs with higher fALFF in the right striatum had shorter MA use and longer abstinence, the increased fALFF in the right striatum might implicate early recovery during abstinence.

## Introduction

Methamphetamine (MA) remains the primary illicit drug threat in China, with over 5 million users in 2018^1^. The use of MA is increasing in parts of Asia and North America, and the number of treatment admissions for MA use disorder also increased in recent years^2^. Chronic MA use has negative consequences on both physical and mental health, which causes a considerable global disease burden^3^. Despite increasing studies on the treatment of MA use disorders, including pharmacotherapy, psychotherapy, and transcranial direct current stimulation, the efficiency in reducing relapse remains unsatisfactory^4–6^.

Relapse is one of the important characteristics of addiction. Indeed, 34% to 77% of patients treated for MA had relapsed at different time points during their treatment programs^7–11^. Even though the overall relapse rate is high, there is evidence suggesting that longer abstinence/treatment period correlates with a lower relapse rate^7,8,10,11^. In China, rehabilitation for drug addiction can be classified as three types: voluntary detoxification, community drug treatment, and incarcerated isolated detoxification. Due to privacy, voluntary detoxification remains the main choice for individuals with MA use disorder. Cumulative evidence shows individuals with addiction have deficits in self-control or self-regulation^12–15^, which is important for regulating habitual behavior. In a forced environment (e.g., hospitalization, incarceration), chronic MA users are abstinent for some time before a relapse opportunity presents. Furthermore, some studies have shown that longer duration of abstinence/treatment predicts lower relapse rates and better performance in decision-making tasks in different substance use disorders^16–18^, with study reported a positive relationship between self-regulation scores and length of abstinence^13^. Taken together, these results indicate the importance of self-control during abstinence and addictive behavior change. According to the self-regulation theory proposed by Baumeister and Heatherton^19^, the ability to effectively regulate behavior is dependent on a limited resource, which is depleted by effortful attempts at self-regulation, leaving individuals more vulnerable to addiction. In an environment where there is no access to addiction drugs, such as a forced environment, less effort is needed to regulate behavior and more resources are retained, which may help individuals resist temptation to drugs when they are discharged.

Resting-state functional magnetic resonance imaging (rs-fMRI) has been used to measure brain activity by detecting low-frequency blood-oxygen-level-dependent (BOLD) signal changes in many psychiatric disorders. Compared to task-based fMRI, rs-fMRI is easily implemented, more tolerable, and captures intrinsic functional brain differences between patients and healthy controls more conveniently^20^. Most existing rs-fMRI studies among MADs exploring resting-state brain activity focused on functional connectivity (FC)^21–23^. FC measures the temporal coincidence of BOLD signals from two spatially distant brain regions, reflecting global characteristics of brain function^24^. While amplitude of low-frequency fluctuations (ALFF) and regional homogeneity (ReHo) are particular methods to reveal different regional characteristics of rs-fMRI data and have been used in many psychiatric disorders^25–28^, but few in addiction^29–31^. ReHo calculates the temporal homogeneity of the BOLD signal between a given voxel with neighboring voxels^32^, reflecting local neural activity while ALFF detects the strength of regional intensity of spontaneous fluctuations in BOLD signal^33^. ALFF may be more effective at measuring local spontaneous activity, and ReHo may be complementary to ALFF in measuring regional abnormalities^34^. While ALFF is sensitive to physiological noise, fractional amplitude of low-frequency fluctuations (fALFF) improves the sensitivity and specificity of spontaneous brain activity detection by measuring the ratio of the power spectrum of low-frequency fluctuations within a specific frequency range to the whole frequency range^35^. Thus, combining ReHo and fALFF to access the spontaneous brain activity among abstinent methamphetamine-dependent subjects (MADs) may provide more information about the underlying brain mechanism during abstinence.

Previous study reported that self-control circuitry mainly included anterior cingulate cortex (ACC), medial prefrontal cortex (mPFC), and striatum^14^. In this study, we aimed to combine ReHo and fALFF to investigate the spontaneous brain activity during resting-state in MADs compared to healthy control subjects (HCs). We hypothesized that the ReHo and fALFF of the resting state would be different between abstinent MADs and HCs in brain areas related to self-control, such as ACC, striatum, and mPFC. We also hypothesized the differences of ReHo or fALFF may relate to the MA use, such as age first started using MA, the duration of MA use, and the duration of abstinence.

## Results

### Demographic characteristic

Twelve participants (5 MADs and 7 HCs) were excluded for analysis due to excessive head motion. A total of 70 MADs and 84 HCs were included in the final analysis. The groups did not differ in gender, age, years of education, or head motion (Table 1). The cigarette smoking per day (CPD) (p < 0.001) and body mass index (BMI) (p = 0.007) in MADs were significantly higher than those in HCs. The MADs had an abstinence period for 38.5 (26.47) days.

**Table 1:** Demographic characteristics of subjects.

	Abstinent MADs (n = 70)	Healthy controls (n = 84)	p
Age (years)*	27 (25,33.00)	29 (26,45)	0.089
Gender (male/female)	59/11	66/18	0.486
Education (years)*	12 (9,14)	12 (9,18)	0.160
BMI*	24.22 (21.73,25.92)	22.41 (20.03,24.64)	0.007
CPD*	20 (10,20)	0 (0,15)	< 0.001
Head motion (mm)^#^	0.112 ± 0.092	0.092 ± 0.049	0.097
Age started using MA (years old)^#^	23.51 ± 5.58	N/A	N/A
Duration of MA use (months)*	60.0 (36.5,76.50)	N/A	N/A
Abstinence from using MA (days)*	38.5 (26.0,47.0)	N/A	N/A

*Median (interquartile range) and Mann–Whitney test are presented.

^#^Mean (standard deviation) and two sample t-test are presented.

### fALFF results between groups

**Figure 1:**
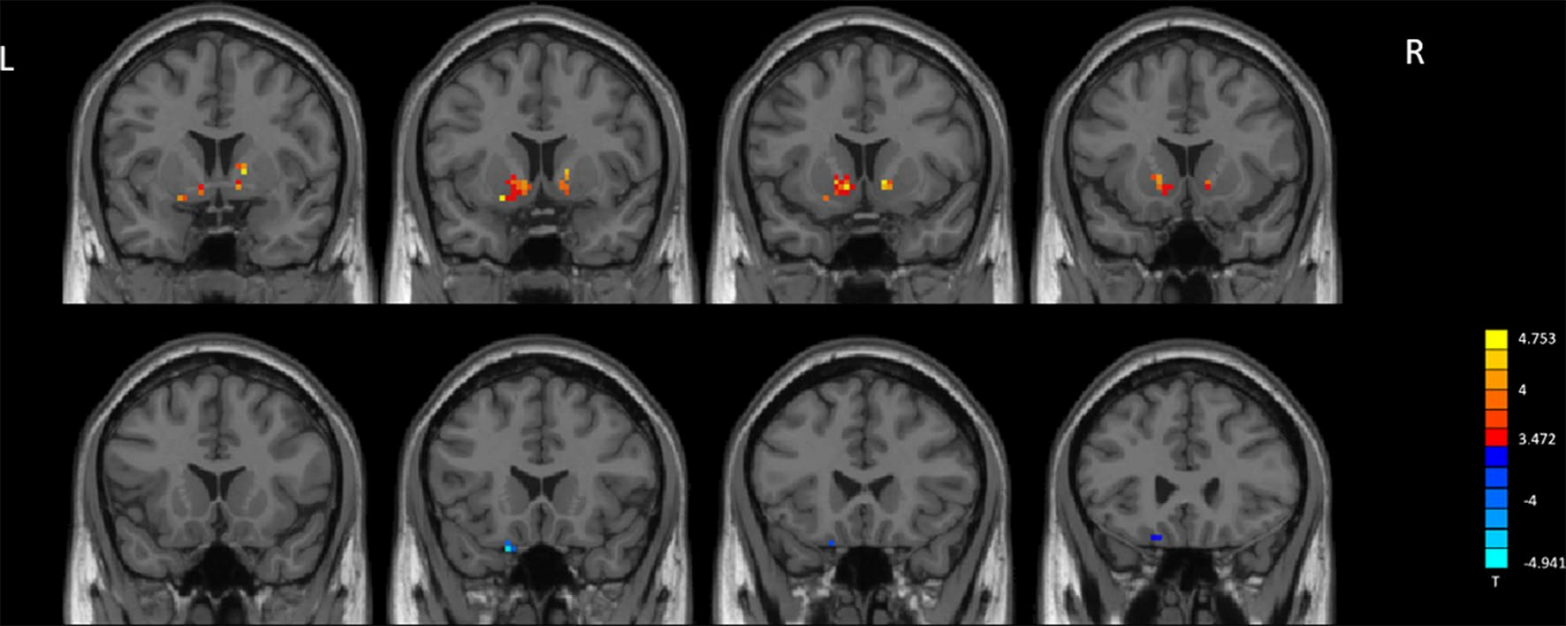
fALFF analysis. Two-sample t-test results are presented, voxel-level p < 0.001, cluster level p < 0.05, two-tailed, voxel size > 9, corrected by GRF. The area in blue represents significantly decreased ALFF value; the area in yellow and red represents significantly increased fALFF value. Created with DPABI_V4.3_200401 (http://rfmri.org/dpabi).

Compared to HCs, MADs showed significantly increased fALFF in the left caudate extending to left putamen, right pallidum extending to right putamen, and right caudate (Fig. 1, Table 2). MADs showed significantly decreased fALFF in left inferior frontal gyrus (IFG) compared to HCs.

**Table 2:** Significant between-group differences in fALFF and ReHo maps.

Condition	Area	Cluster	T (peak)	Peak MNI coordinates (mm)		
				x	y	z
**fALFF**						
MADs > HCs	Left caudate/putamen	39	4.7529	− 9	12	− 6
	Right pallidum/putamen	19	4.6989	15	6	3
MADs < HCs	Left inferior frontal gyrus	11	− 4.9418	− 18	21	− 27
**ReHo**						
MADs > HCs	Left caudate/putamen	55	5.5531	− 9	12	− 6
	Right putamen/caudate	65	5.315	15	12	− 3
MADs < HCs	Right postcentral gyrus/precentral gyrus	237	− 5.3121	57	− 6	24
	Bilateral anterior cingulate	29	− 4.027	3	36	12
	Left postcentral gyrus/precentral gyrus/precuneus	182	− 4.6663	− 42	− 36	51
	Left postcentral gyrus	30	− 4.191	− 54	− 9	24

*MADs* methamphetamine-dependent subjects, *HCs* health control subjects, *MNI* Montreal Neurological Institute.

### ReHo results between groups

**Figure 2:**
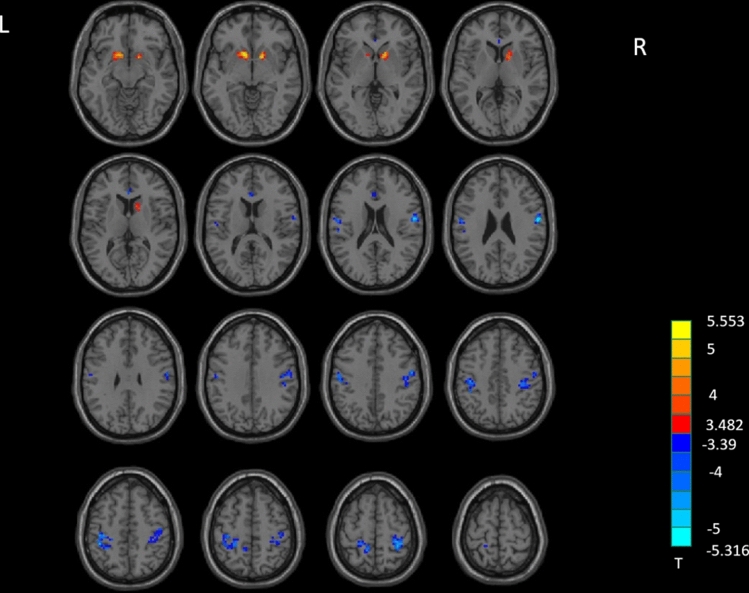
ReHo analysis. Two-sample t-test results are presented, voxel level p < 0.001, cluster level p < 0.05, two-tailed, voxel size > 29, corrected by GRF. Area in blue with significantly decreased ReHo value; area in yellow and red with significantly increased ReHo value. Created with DPABI_V4.3_200401 (http://rfmri.org/dpabi).

MADs showed significant increased ReHo in the left caudate extending to left putamen, and the right putamen extending to right caudate, and decreased ReHo in the bilateral anterior cingulate cortex (ACC), right postcentral gyrus extending to right precentral gyrus, and the left postcentral gyrus extending to left precentral gyrus and left precuneus, compared to HCs (Table 2, Fig. 2).

### Regression and correlation analysis results

**Figure 3:**
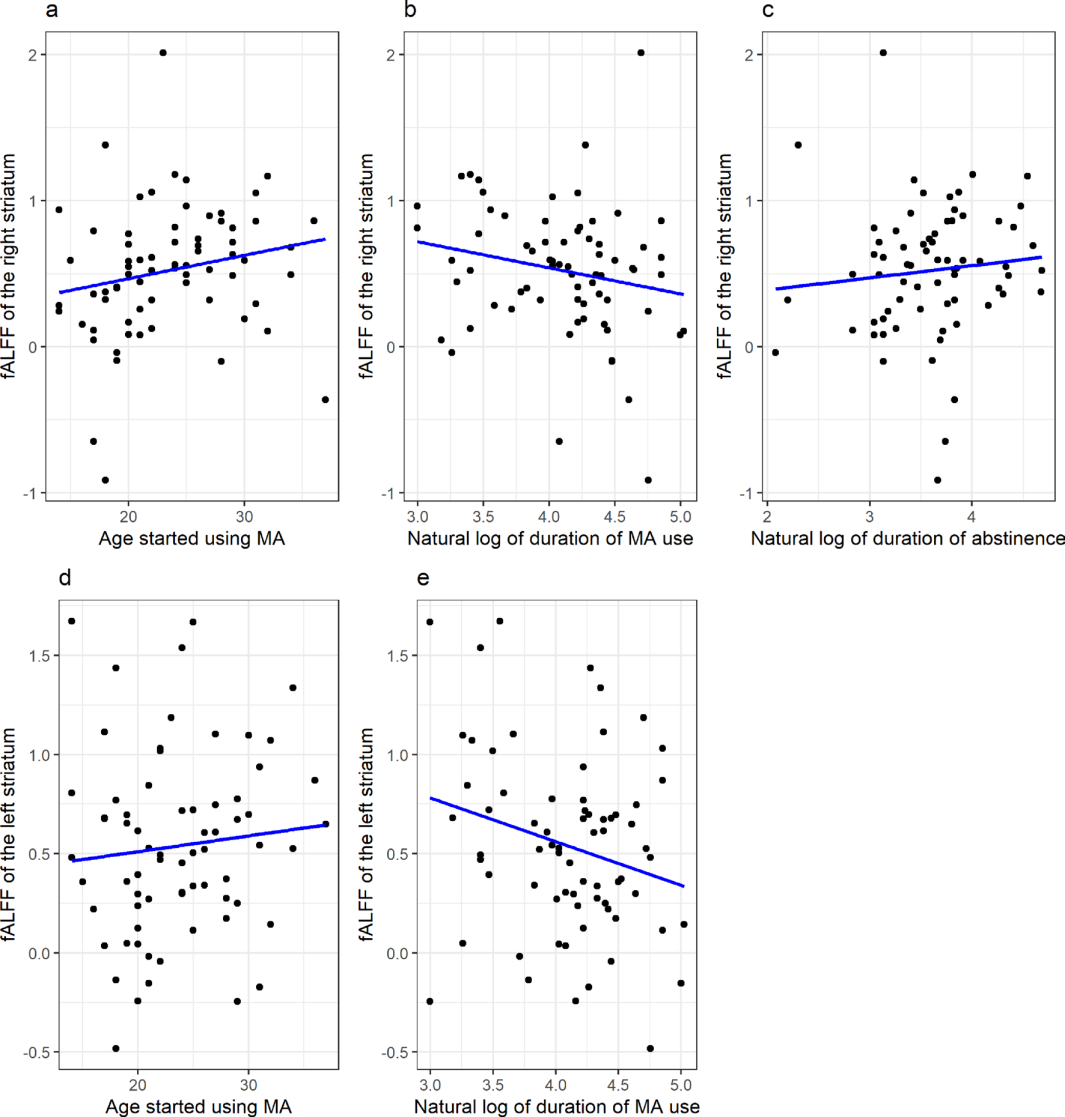
Correlation figures. (**a**) Relationship between the fALFF value of the right striatum and the age started using MA. (**b**) Relationship between the fALFF value of the right striatum and the natural log of duration of MA use. (**c**) Relationship between the fALFF value of the right striatum and the natural log of duration of abstinence. (**d**) Relationship between the fALFF value of the left striatum and the age started using MA. (**e**) Relationship between the fALFF value of the left striatum and the natural log of duration of MA use.

fALFF of the right striatum (adjusted R^2^ = 0.231, *p* = 0.002) negatively correlated with natural log of duration of MA use (B = -0.156, *p* = 0.044), positively correlated with age started using MA (B = 0.020, *p* = 0.024) and natural log of duration of abstinence (B = 0.161, *p* = 0.035) (Table 3, Fig. 3). fALFF of the left striatum (adjusted R^2^ = 0.157, *p* = 0.029) negatively correlated with the natural log of the duration of MA use (B =  − 0.268, *p* = 0.035), and positively correlated with age started using MA (B = 0.023, *p* = 0.035). Model p values were corrected with Bonferroni correction and predictor p values were corrected with BH procedure. A similar pattern was observed in the left IFG; however, it did not survive corrections for multiple comparisons. Other results were not significantly but they were reported in the Supplementary Materials.

**Table 3:** Regression results.

Regression variables	Regression 1: fALFF of the right striatum			Regression 2: fALFF of the left striatum		
	B	Raw p-value	Corrected p-value	B	Raw p-value	Corrected p-value
Natural log of duration of MA use	− 0.156	0.037	0.044	− 0.268	0.012	0.035
Age started using MA	0.020	0.004	0.024	0.021	0.023	0.035
Natural log of duration of abstinence	0.161	0.022	0.035	0.107	0.268	0.268

## Discussion

This study is the first to compare the fALFF and ReHo differences between abstinent MADs and HCs in a large sample. Compared to HCs, abstinent MADs showed significantly increased fALFF and ReHo in the bilateral striatum (caudate and putamen), decreased fALFF in the left IFG and decreased ReHo in the bilateral ACC, precentral and postcentral gyrus, and left precuneus. Furthermore, fALFF values of the right striatum cluster and the left striatum cluster were negatively correlated with the natural log of the duration of MA use and positively correlated with age started using MA, with fALFF value of the right striatum cluster was positively correlated with the natural log duration of abstinence.

A few of studies with a small sample size used a single method (ReHo^36–38^ or ALFF^39^) to explore resting state brain activity among MADs. Some studies had combined ALFF and ReHo among individuals with psychiatric disorders^40–42^ and other addiction disorders^43,44^, but not in MADs. As fALFF and ReHo could be complementary to each other, combining these two methods to explore resting state brain activity among a relatively large sample of MADs may reveal more information regarding to the pathophysiological framework in the brain during abstinence.

In this study, increased fALFF and ReHo in the bilateral striatum for MADs may indicate higher amplitude of BOLD signals and increased spontaneous regional neural activity in these two brain regions. The altered fALFF and ReHo in the bilateral striatum not only revealed abnormal and non-synchronous spontaneous neural activity in these two regions but also highlights them as core brain regions that are altered in the resting brains of abstinent MADs. Only a few rs-fMRI studies have investigated the alternation of spontaneous brain activity and synchronization using fALFF or ReHo among individuals with addiction disorders. Active heroin users showed decreased ALFF in the right caudate, which was negatively correlated with the duration of heroin use^31^. A study compared heroin users with and without relapse found that relapsed heroin users had increased ReHo in the right caudate^45^. Among individuals with internet gaming disorder, ALFF values of the bilateral putamen were higher before cognitive based treatment (CBT), while ALFF of left putamen was lower after CBT compared to HCs^46^. Chronic active smokers showed significantly increased fALFF in the right caudate, relative to HCs^47,48^. Although our results are not quite consistent with these studies, which may be due to the heterogeneity of samples (i.e., different addiction, different state of patients at data acquisition, and different sample size), these results consistently indicate the bilateral striatum as key areas in addiction disorders, in line with previous studies. Furthermore, the values of fALFF and ReHo may change during the course of abstinence. Both addictions and obsessive–compulsive disorder (OCD) show abnormalities in striatum, which is related to impaired self-control^49^. OCD patients exhibited lower ReHo in the bilateral caudate before treatment and increased ReHo in the left caudate after CBT, along with the clinical improvement^50^. Abnormalities such as enlarged volumes^51,52^, lower metabolism^53,54^, down-regulation of levels of stored dopamine^55^, dopamine release, dopamine transporter and receptor availability in the striatum^56,57^ in abstinent MADs have been reported in previous studies. These findings revealed the important roles of striatum in the pathology of MA dependence. Since these results are observed during abstinence, some of the neurotoxic effects of MA appear to be persistent, while some studies reported partial recovery in MADs with protracted abstinence^54^, some even with rapid recovery^55^. Thus, the increased fALFF and ReHo values of bilateral striatum in the present study may indicate partial recovery of striatum function in abstinent MADs.

Importantly, we found that the fALFF value of the right striatum was positively correlated with duration of abstinence, which may suggest that long-term abstinence is beneficial for the recovery of striatum function, such as self-control. MADs living in an environment where there is no access to MA do not have to make as many effort as those who have access to MA, thus more resources retained for better self-control^19^, which may be related to the increased fALFF value of the striatum. Moreover, the fALFF values of bilateral striatum were negatively correlated with the duration of MA use, which may further suggest that the increased fALFF values of bilateral striatum, especially the right striatum, are associated with abstinence, or even early recovery, rather than only MA impairments. Within this context, the present findings suggest that the fALFF and ReHo values of the bilateral striatum change over the course of abstinence. The fALFF values of bilateral striatum may decrease during active MA use and early stages of abstinence, possible due to MA acute effects, then increase with longer periods of abstinence due to partial recovery or a compensatory reaction. Thus, fALFF values of bilateral striatum, especially the right striatum, may be sensitive to the change of state between active use and abstinence. While the fALFF and ReHo values of bilateral striatum may not continually increase or even may decrease to levels the same as healthy controls at some timepoint, this is only a speculation as the current study did not have a long abstinent follow-up data to test this. Further work is needed to demonstrate these dynamic changes.

Adolescents are more vulnerable to drug use-related alterations than adults^58–60^. Animal studies showed a similar pattern whereby the younger the age of exposure to MA, the more vulnerable and long-lasting the impairment is^61,62^. These findings are largely consistent with our present results, in which the increased fALFF values of bilateral striatum were positively correlated with the age of first MA use. This indicates that the younger the age of first MA use, the less the fALFF recovery during abstinence.

In our study, MADs showed decreased fALFF in the left IFG. The IFG is thought to play an important role in response inhibition^63^. Both MADs and individuals with other addiction disorders exhibited lower activation in IFG during response inhibition-related task and decision-making task^64–66^, which correlated with impairment of inhibition control. Decreased grey matter volume of the left IFG was found in both MADs^67^ and alcohol-dependent patients compared to HCs^68^. Moreover, early abstinent MADs had reduced cerebral blood flow (CBF) of the IFG^69^. A previous study demonstrated both ReHo and ALFF are reliably correlated with regional CBF in most brain regions^70^. Therefore, decreased fALFF in the left IFG is suggestive of MA-related impairment.

The decreased ReHo in the bilateral ACC in the present study is consistent with previous studies among abstinent MADs^38^, alcohol use disorder subjects^71^, and betel quid dependence subjects^43^, compared to HCs. ACC is recognized as an important area in addiction. Previous studies of MADs have shown decreased glucose metabolism^72^, abnormal metabolite levels^59^, decreased cerebral blood flow^73^, and hypoactivation during decision-making tasks^74,75^, which are associated with deficits in attentional control, cognitive functions of behavior monitoring, risk-related processing, and self-control^14^. ACC is actively involved in cognitive control, emotional regulation, and self-control^76^. These results lead us to speculate that the functional changes in the ACC may underlie impaired cognitive function and self-control in MADs.

As a key functional region of the DMN^77,78^, the precuneus is involved in the awareness of the perception of environmental stimuli (exteroception)^79^. Exteroception contributes to hyper-sensitivity to self-relevant external cues associated with their drug use in addiction. In alignment with this theory, increased precuneus activation when exposed to drug cues is widely-reported in different addicted populations^80–82^, which elicit craving related to cue reactivity. Precuneus has been considered as a vulnerable region in addiction as it is a core region of the exteroception network^79^. For example, individuals with MA associated psychosis showed stronger ReHo in the right precuneus than individuals with schizophrenia^37^. The inconsistency with our present result is reasonable due to different samples. In addition, decreased glutamate + glutamine was observed in the precuneus in early abstinent MADs^83^. Cortical thickness trended smaller in precuneus in MADs^84^. Consequently, we speculate that the decreased ReHo in the left precuneus is related to dysregulation of the exteroception process due to MA use.

It is noteworthy that decreased ReHo was found in the bilateral precentral gyrus and postcentral gyrus, i.e., the sensorimotor cortex. The effects of drug exposure on the sensorimotor cortex have not been fully investigated yet. While smokers showed fALFF decreased left postcentral gyrus^85^, MADs showed decreased glucose metabolism^72^ and lower grey matter volume in the precentral gyrus^86^. Although few studies have looked specifically at MA effects within the sensorimotor cortex, they have extensive connections. Previous studies report putamen-sensorimotor circuits play an important role in habitual responding^87^ and relapse^88^. The abnormal ReHo values of bilateral precentral gyrus and postcentral gyrus may suggest that these regions play an important role in MA-related impairment.

There are several limitations in the neuroimaging study. First, this is a cross-sectional study, so we could not confirm the causal relationship between these alterations with MA consumption, or abstinence, or a combination of MA consumption and abstinence. Second, we focused on resting state and did not apply any cognitive tasks, so we could not show whether brain alterations were correlated with cognitive impairment. Third, the MADs and healthy controls were not matched exactly regarding BMI or CPD. During the abstinence, MADs could not access to MA, and may lead to withdrawal symptoms like anxiety; many would increase cigarette use, sleep a lot, decrease physical activity, and eat more snacks, which may increase body weight. Therefore, both BMI and CPD of MADs were higher than HCs. Although both CPD and BMI showed influence in ReHo and fALFF in several brain regions^47,85,89–91^, these alterations could not explain all the results in our study. Furthermore, we included BMI and CPD as covariates to control these effects in the analysis. Finally, most of the subjects were male, a possible gender difference was unexplored. Therefore, future studies should recruit more female MADs, match subjects’ CPD, and BMI, include cognitive function tests, and measure alterations during different stages (i.e., active MA use, early abstinence, and long-term abstinence).

Nevertheless, this first study showed abstinent MADs had abnormal resting-state function in cortical and subcortical regions in bilateral striatum, left IFG, bilateral ACC, precentral gyrus, postcentral gyrus, and left precuneus in a large sample. These brain alterations may relate to self- and cognitive control and exteroception. We also observed increased fALFF in striatum were correlated with duration of MA use, age of first MA use and duration of abstinence. We speculate that the decreased fALFF and ReHo may be the result of MA consumption and the increased fALFF and ReHo in the bilateral striatum may result from abstinence. These findings may help to elucidate the pathophysiology in MADs.

## Methods

### Subjects

The data were collected as a part of the brain imaging study on methamphetamine-induced psychotic symptoms, a study hosted at the Hunan Provincial People’s Hospital and the Second Xiangya Hospital of Central South University. One hundred and sixty-six subjects (75 MADs and 91 drug-free HCs matched for age, gender and education level, age 18–45) were enrolled in this study. MADs were recruited from the Kangda Voluntary Drug Rehabilitation Centers in Hunan Province. All MA users fulfilled the Diagnostic and Statistical Manual of Mental Disorders, fourth edition (DSM-IV)^92^ criteria for lifetime MA dependence assessed by the Structured Clinical Interview (SCID)^93^. MADs were excluded if they met criteria for other substance dependence (excluding nicotine dependence) at any time. Subjects were required to abstain from MA for at least 48 h before scanning. Drug-free HCs were recruited from the community through advertising. Participants were excluded if they (i) had any general medical condition or neurological disorders, including infectious, hepatic, or endocrine disease; (ii) had a history of severe head injury with skull fracture or loss of consciousness of more than 10 min; (iii) had any current or previous psychiatric disorder; (iv) had a family history of psychiatric disorder; (v) women during pregnancy or breast-feeding stage; (vi) had contraindications for MRI. Two licensed psychiatrists, at MD level, conducted all clinical interviews. Subjects were fully informed about the measurement and MRI scanning in the study. Written informed consent was given by all subjects. This study was approved by the Ethics Committee of the Second Xiangya Hospital, Central South University (No. S095, 2013), and was carried out in accordance with the Declaration of Helsinki. The demographic characteristics are shown in Table 1.

### Magnetic resonance data acquisition

The images were acquired using standard sequences with a Siemens Magnetom Trio 3.0 T MRI scanner (Siemens, Erlangen, Germany) equipped with an eight-channel head coil at the Magnetic Resonance Center of Hunan Provincial People’s Hospital, China.

Three-dimensional T1-weighted structural brain images were acquired with a gradient echo sequence: repetition time = 2,000 ms, echo time = 2.26 ms, field of view = 256 × 256 mm, flip angle = 8°, matrix size = 256 × 256, number of slices = 176, slice thickness = 1 mm.

The functional images were collected using an echo-planar imaging sequence with the following parameters: number of slice = 32, repetition time = 2,000 ms, echo time = 30 ms, slice thickness = 4.00 mm, flip angle = 90°, matrix = 64 × 64, field of view = 220 × 220 mm^2^.

### Data preprocessing

Functional and structural images were processed by Data Processing & Analysis of Brain Imaging (DPABI)^94^ using Data Processing Assistant for Resting-State fMRI (DPARSF)^95^. The first ten volumes were discarded to allow for signal stabilization and subjects adaptation. The remaining volumes were corrected for slice time differences, realigned to correct for small movements, and corrected for head motion. Subjects with head motion exceeding 2.0 mm in any dimension or 2° of any angular rotation were excluded from further analysis. Individual functional images were then coregistered to T1-weighted MR images, which were segmented and normalized to the standard structural MRI template in the Montreal Neurologic Institute space using nonlinear transformation. Spatially normalized images were then detrended to remove linear trends and remove nuisance signals, including white matter, cerebrospinal fluid signals, mean global signal, and 24 motion parameters.

### Calculation of fALFF and ReHo

fALFF and ReHo values were calculated based on previous studies^32,33^ using DPABI. For fALFF analysis, the detrended functional images were smoothed with a Gaussian kernel of 4 mm full-width at half-maximum (FWHM). Power spectrum were computed by transforming time series of each voxel to the frequency domain via Fast Fourier Transform. The average square root of the power spectrum at each voxel across 0.01–0.1 Hz was taken as the ALFF. fALFF was obtained as the division of ALFF by the whole frequency range observed in the signal.

ReHo calculation was performed on a voxel-by-voxel basis by calculating Kendall’s coefficient of concordance (KCC) of the time series of a given voxel with its nearest 26 voxels^32^. Then the individual ReHo maps were smoothed with a Gaussian kernel of 4 mm FWHM.

A whole-brain mask was used to remove the nonbrain tissues. Prior to subsequent analyses, individual fALFF and ReHo maps were standardized into z-score maps by dividing the global mean fALFF and mean KCC within the whole-brain mask.

### Statistical analysis

Statistical analysis was performed in R 3.6.1 within Rstudio^96^. Differences between MADs and HCs in demographic variables, i.e., age, gender, duration of education, marriage, CPD, and head motion were tested using two-sample t-tests, Mann–Whitney tests, and Pearson’s chi-squared tests using R, and a p < 0.05 was set as significant.

Analysis of fALFF and ReHo maps were performed with voxel-wise two-sample t-tests in DAPBI. We included age, CPD, BMI, head motion, and grey matter volume as covariates. Significant differences in the analysis were reported using the criteria of multiple comparisons with the Gaussian Random Field theory correction (GRF) (voxel-wise p < 0.001, cluster-wise p < 0.05, two-tailed) and with a minimum extent threshold of 30 voxels for ReHo, 10 voxels for fALFF.

Regions where the MADs showed significant differences over the HCs for ReHo or fALFF properties were determined as regions of interest (ROIs). ReHo/fALFF values of these regions were extracted, averaged, and regressed against MA use parameters (i.e., age started using MA, duration of MA use, duration of abstinence). MA use parameters that were not normally distributed were natural log-transformed for the regression analyses^51^. We corrected the model p value for each regression model by using Bonferroni correction^97^. Models with corrected p value less than 0.05 would be considered as there was a significant relationship between the value of fALFF or ReHo and MA use parameters. Then Benjamini–Hochberg (BH) procedure was applied to control the false discovery rate (FDR)^98^ to correct the p value of MA use parameters in the models with corrected model p value less than 0.05.

## Supplementary Information

Supplementary Information.
