# Supplementary material for: Altered patterns of fractional amplitude of low-frequency fluctuation and regional homogeneity in abstinent methamphetamine-dependent users
Source: Sci Rep. 2021 Apr 8;11:7705. doi: 10.1038/s41598-021-87185-z (PMC8032776; doi:10.1038/s41598-021-87185-z)
Supplement: Supplementary file 1 — Supplementary Information. [file 41598_2021_87185_MOESM1_ESM.pdf]

Altered patterns of fractional amplitude of low-frequency fluctuation and regional homogeneity in abstinent methamphetamine-dependent users

An Xie[1#], Qiuxia Wu [2,3#], Winson Fu Zun Yang[3], Chang Qi[4], Yanhui Liao[5], Xuyi Wang[2], Wei Hao[2], Yi-Yuan Tang[3], Jianbin Liu[1*], Tieqiao Liu[2*] and Jinsong Tang[5]

1. Department of Radiology, Hunan Provincial People's Hospital, First Affiliated Hospital of Hunan Normal University, Changsha 410005, China.

2. National Clinical Research Center for Mental Disorders, and Department of Psychaitry, The Second Xiangya Hospital of Central South University, Changsha 410011, Hunan, China.

3. Department of Psychological Sciences, Texas Tech University, Lubbock, TX, United States.

4. Department of Psychiatry, Zhejiang Provincial People's Hospital, Department of Psychiatry, People's Hospital of Hangzhou Medical College, Hangzhou, China.

5. Department of Psychiatry, Sir Run Run Shaw Hospital, School of Medicine, Zhejiang University, Hangzhou, Zhejiang, China.

# These authors contributed equally to this manuscript

* These authors jointly supervised this work.

Correspondence to:

Jianbin Liu, Department of Radiology, Hunan Provincial People's Hospital, First Affiliated Hospital of Hunan Normal University, Changsha 410005, China. Email: binban24@163.com

Tieqiao Liu, National Clinical Research Center for Mental Disorders, and Department of Psychaitry, The Second Xiangya Hospital of Central South University, Changsha 410011, Hunan, China. Email: liutieqiao123@csu.edu.cn

Table S1. Other regression results

	Natural log of duration of MA use		Age started using MA		Natural log of duration of abstinence	
	B	P	B	P	B	P
Regression 1: ReHo of right striatum	-0.075	0.365	0.001	0.354	0.023	0.759
Regression 2: ReHo of left striatum	-0.002	0.087	0.011	0.092	0.002	0.309
Regression 3: ReHo of ACC	0.011	0.925	-0.011	0.278	0.009	0.933
Regression 4: ReHo of right sensorimotor cortex	-0.047	0.385	-0.006	0.209	-0.015	0.770
Regression 5: ReHo of left sensorimotor cortex	-0.005	0.926	-0.005	0.273	-0.001	0.980
Regression 6: ReHo of left postcentral gyrus	0.009	0.919	0.001	0.922	0.091	0.261
Regression 7: fALFF of left IFG*	0.026	0.818	0.023	0.021	0.009	0.405

*The whole regression model is not significant.
